# Do You Feel the Same as I Do? Differences in Virtual Reality Technology Experience and Acceptance Between Elderly Adults and College Students

**DOI:** 10.3389/fpsyg.2020.573673

**Published:** 2020-09-25

**Authors:** Qian Liu, Yanyun Wang, Qingyang Tang, Ziwei Liu

**Affiliations:** ^1^School of Journalism and Communication, Beijing Normal University, Beijing, China; ^2^Institute of Communications Research, College of Media, University of Illinois Urbana-Champaign, Champaign, IL, United States

**Keywords:** virtual reality, technology acceptance, media experience, age differences, elderly, college students

## Abstract

Virtual reality (VR) has been widely applied in medical health areas and the entertainment and tourism industries, in which elderly people are a primary target. However, most studies on VR have focused on how people respond to this new technology and its effects on younger generations. Our study explored the differences in VR experience and acceptance between elderly adults and college students. A mixed method approach including both experiments and in-depth interviews was applied in this study. Our results show that elderly adults reported a higher level of telepresence and stronger emotional responses than young adults in VR viewing regardless of the VR device. The ways in which elderly people processed and evaluated VR content and devices were different from those of young people. Elderly people reported more positive attitudes toward the video they watched than young people, but this did not necessarily lead to more positive attitudes toward the viewing experience.

## Introduction

Virtual reality (VR) technology has been applied in various fields, such as news (La Peña et al., 2010), brand marketing and advertising (Coyle and Thorson, 2001; Klein, 2003; Hopkins et al., 2004; Fiore et al., 2005), tourism promotion (Guttentag, 2010; Huang et al., 2013), games and entertainment (Faiola et al., 2013), education (Roussou, 2004) and medical health (Bush, 2008; Garrett et al., 2014; Amin et al., 2017; Hsieh and Lee, 2018). In recent years, VR has become a powerful and popular tool for creating immersive experiences for brand marketing and advertising (Shen et al., 2019). VR technology has emerged in commercial activities, such as information dissemination, advertising and sales (Barnes, 2016). According to a business news report, 75% of the world’s most valuable brands (such as Apple, Google, Coca-Cola, and Microsoft) have created VR projects to attract consumers (Korolov, 2015). VR technology also provides a variety of applications in tourism policy planning, tourism marketing, tourism attractions, entertainment and heritage conservation (Guttentag, 2010). VR has the potential to provide an immersive emotional experience in travel, including in urban and architectural landscapes, specific destinations, museum items, etc. (Barnes, 2016). Marriott Hotels uses VR to promote Hawaii honeymoon experiences, in which visitors can walk around the virtual environment, view the scenery, and enjoy the sensory experience of fog and warm air (Huang et al., 2013). Previous research on the effectiveness of VR technology has achieved significant and promising results. Immersive VR goggles generate more telepresence than a traditional 2D broadcasting device (Shen et al., 2019), and the influence of telepresence in enhancing consumers’ beliefs and attitudes toward brands, products and advertisements has been experimentally confirmed (Coyle and Thorson, 2001; Klein, 2003; Hopkins et al., 2004; Fiore et al., 2005). Nevertheless, most of these studies have focused on how people respond to this new technology and its effects on younger generations, but we do not know whether these effects exist in all age groups (Liu et al., 2020).

VR technology has been shown to be an effective health treatment approach and has achieved remarkable results in distraction from acute pain, the treatment of mental disorders and medical training (Amin et al., 2017). Herrero et al. (2014) asked patients with fibromyalgia to use VR goggles to view pictures with language guidance and found that the subjects reported significantly increased self-efficacy, increased positive emotions, decreased negative emotions and decreased pain caused by the disease. The sense of telepresence generated by VR can trigger different emotions, which is related to the emotional information provided by the application scenarios and specific virtual scenes. Felnhofer et al. (2015) created five VR park scenes with different emotional properties and successfully induced the corresponding emotions in participants. VR was found to be an effective tool for inducing emotions and has been applied to treat mental diseases. Stupar-Rutenfrans et al. (2017) significantly reduced the level of individuals’ public speaking anxiety with virtual reality exposure therapy, and the effect was better for participants with higher initial anxiety levels. In addition to exposure therapy, mindfulness training has also been shown to be a therapeutic method combined with VR, and the combination has reduced negative emotions such as depression and anxiety, especially for groups with emotional problems (Flores et al., 2018). Evans et al. (2020) confirmed the better acceptability and effectiveness of personalized virtual reality experience therapy than other forms of therapy in promoting healthy emotions, and the degree of positive emotional improvement was related with personal characteristics and the initial emotional status. In addition, VR technology can also provide virtual anatomy and virtual operating tables to improve traditional medical teaching methods and medical nursing training by allowing trainees to interact with VR and reduce technical operating mistakes caused by negligence (Hsieh and Lee, 2018).

Moreover, researchers have conducted trials by applying VR technology to improve elderly adults’ mental and physical well-being. To help elderly patients with impaired cognitive function problems, researchers designed a special VR therapy program for elderly patients to perform daily routines, and the program achieved allowed elderly patients to achieve significant improvements (Gamito et al., 2019). Ten veterans of the Vietnam War diagnosed with fourth-stage posttraumatic stress disorder received 10 VR therapy treatments (90 min each time), and in their 6-month follow-up assessments, their symptoms of posttraumatic stress disorder were significantly reduced (Rothbaum et al., 2001). Levy et al. (2016) designed a virtual reality game to treat the phobia of falling among elderly people. The results showed that this intervention had a significant effect on reducing their anxiety about falls.

In conclusion, VR has been studied in different areas with specific age groups. Since VR is becoming more prevalent as a greater variety of content is available to audiences and as the cost of devices is decreasing, VR has become widely applied in many fields including medical health areas and the entertainment and tourism industries, where elderly people can be a primary target, there is a need to study VR as a daily use digital media device similar to smartphones and computers. Huygelier et al. (2019) found that elderly participants’ initial attitudes toward VR were neutral before experience and turned to be positive after the first-time exposure. After using VR for 15 min twice a week for 6 weeks, those elderly participants showed high acceptance of VR and reported positive perceptions toward adopting VR (Syed-Abdul et al., 2019). A recent review study on applying immersive VR technology to the elderly proposed an immersive experience model of 20 application methods and suggested that such applications could be expanded to various fields like entertainment, education, and media in the elderly welfare centers (Lee and Park, 2020).

It was not surprising to find age group differences in acceptance and experience with new technologies. An intra-subject design study revealed significant age-related differences (seniors above 60 years old vs. adults up to 40 years old) across head-mounted display vs. desktop platforms in both assessed performance and user experience after a virtual supermarket shopping task (Plechatá et al., 2019). In terms of emotion effects, an experimental study revealed differences in the processing of positive and negative emotions between age groups (seniors above 60 years old vs. college students) after watching a 360-degree video by a VR headset or an iPhone (Liu et al., 2020). However, studies that directly compare the two age groups (young people vs. elderly people) using the same VR stimuli and that explore whether they process the experience differently were still very few and lack of particulars.

The current study aims to explore the experience and acceptance differences between elderly adults and college students from the following aspects:

To evaluate the experience and acceptance difference levels in terms of perceived telepresence, video preference, viewing experience and purchase intention between the two age groups;

To test the mechanism of how telepresence, video preferences and viewing experiences work in different age groups;

To further explore the details and reasons behind those differences by qualitative in-depth interviews.

## Materials and Methods

### Study Design and Materials

The study was conducted among two age groups (i.e., elderly adults aged 60 and above and college students) during 1 week of April 2019. The two experimental groups were subjected to the same study design, procedures and measurements. A prepost treatment between-subjects design was applied in our study. The participants in the elderly group and the college student group were randomly assigned to either the VR condition (Pico 4K G2) or the smartphone condition (iPhone 8) to watch the same video, named VR China, which was an 8-min scenery documentary provided by National Geographic (China). The study received university IRB approval.

### Participants

The elderly participants in the study were recruited from a community service center in Beijing, China. The research notice was sent out to all the residents of the community through WeChat. Eventually, 58 elderly adults aged 60 and above signed up, showed up and completed all the procedures in our study. Their ages ranged from 60 to 91 years old (M = 68.84, SD = 7.095). There were 36 female and 22 male participants.

To recruit the young participants (i.e., college students), a group of university staff and students helped distribute the research notice to university students through WeChat. Sixty student participants, including 30 females and 30 males, completed the study. Among them, the youngest was 18 years old, and the eldest was 25 years old (M = 20.38, SD = 2.164).

### Procedure

In this study, it took approximately 1 h to complete the 5-step experiment with the assistance of research assistants. Step 1: An informed consent form was prepared for each participant to read and sign before the experiment. Step 2: Each participant completed a pretest questionnaire containing several questions related to his or her previous VR experience, preferences, and attitudes toward VR. Step 3: Each participant watched an approximately 8-min stimulus video in Chinese voice-over with Chinese subtitles using a smartphone or wearing a VR headset. Step 4: The participants were asked to complete the postquestionnaire. Step 5: The research assistant conducted a semistructured interview with each participant for approximately 30 min. Part 1 of the study (for the elderly group) was conducted in the community service center rather than in the lab due to the consideration of the possible health and safety issues of the elderly participants during the transportation and experimental period. For the elderly participants who had difficulty reading, the research assistants read aloud and explained the information on the consent form and the pre- and postsurveys. Part 2 (for the college student group) was conducted with the college students in a lab at a university in Beijing.

An approximately 7-dollar cash coupon was given as a reward to each participant who completed the study. In addition, we asked those who were assigned to the non-VR condition whether they wanted to experience the VR viewing after the experiment.

### Dependent Measures

#### Perceived Telepresence

Telepresence was defined as a psychological state of presence brought by media content such as movies or TV dramas (Kim and Biocca, 1997; Suh and Lee, 2005), which was enhanced by the immersive VR technology (Biocca, 1997; Rupp et al., 2016; Van Damme et al., 2019). Participants’ perceived telepresence while watching the video stimuli was measured on a 7-point scale adopted from Kim and Biocca (1997), with 1 being “strongly disagree” and 7 being “strongly agree.” Participants were asked to what degree they agreed on the eight statements like “During the viewing experience, I felt I was in the world the television created.” The average score was calculated as the overall telepresence score for each subject (some items were reverse coded).

#### Video Preference

Participants’ preferences regarding the video were also measured in the questionnaire by asking to what extent they liked the video in general, with 1 being “dislike a lot” and 7 being “like a lot.”

#### Viewing Experience

Participants’ attitudes toward the viewing experience were measured by asking to what extent they liked the way they viewed the video, with 1 being “dislike a lot” and 7 being “like a lot.”

Both video preference and viewing experience were measured by a single-item question as the two constructs are easily and uniformly understood (Bergkvist and Rossiter, 2007). Besides, a single-item measure can avoid the bias of the common methods (Williams et al., 1989).

#### Purchase Intention (PI)

Whitlark et al. (1993) defined purchase intention as a purchase probability associated with an intention category at the percentage of individuals that would buy a product. Similar to a measure used in Kergoat et al. (2017), purchase intention was measured on a 5-point Likert scale two times (Bearden et al., 1984), i.e., once before and once after people watched the video, by asking the participants to what extent they would like to purchase a VR headset, with 1 being “would like very much” and 5 being “would not like very much.”

#### Qualitative Data

The data from semistructured interviews were also recorded for further analysis.

## Results

### Quantitative Results

Previous studies have found that males and females differ in the ways they experience VR and in the levels of perceived telepresence. To avoid potential gender differences that would bias the results, a chi-square test was conducted to check whether male and female participants were randomly assigned to the VR and smartphone before performing the main analyses. For the elderly individuals, there was no significant difference in the gender distribution between the two conditions [χ^2^ (1, 58) = 2.78, *p* = 0.096]. For the college students, there was also no significant difference in the gender distribution between the two conditions [χ^2^ (1, 60) = 2.40, *p* > 0.5]. Therefore, gender differences should not have affected the results of our study. Besides, we conducted Levene’s test to check the homogeneity of variance. The results showed that the Levene Statistics of perceived telepresence [Levene(1,116) = 0.202, *p* > 0.05], video preference [Levene(1,116 = 2.487, *p* > 0.05], and viewing experience [Levene(1,116) = 1.646, *p* > 0.05] in our study were all non-significant. Therefore, equal variances can be assumed for the following ANOVA analyses.

#### Perceived Telepresence

**FIGURE 1:**
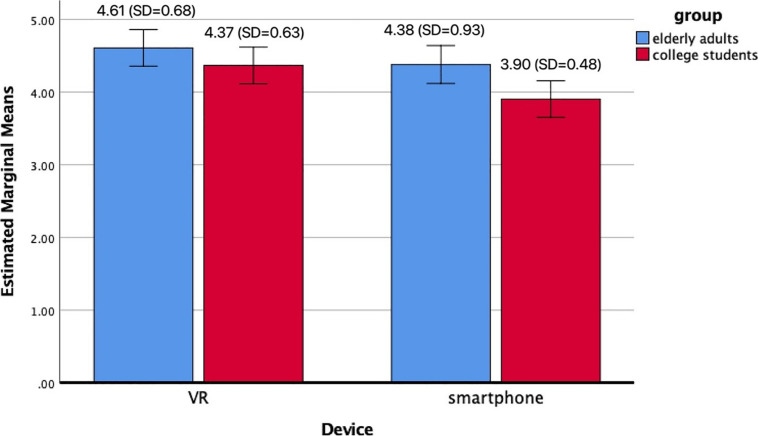
Effects of age group and device on perceived telepresence.

To explore the differences in the perceived telepresence in the two age groups (college students vs. elderly adults), a two-way ANOVA was conducted using the telepresence level as the dependent variable and age group (undergraduate students vs. elderly adults) and device (VR vs. smartphone) as the independent variables (see Figure 1). The results showed significant main effects of both the age [*F*(1,116) = 7.80, *p* < 0.01] and device [*F*(1,116) = 7.22, *p* < 0.01] on perceived telepresence. Regardless of age, viewing the video in an immersive VR environment elicited a higher level of perceived telepresence (M = 4.5, SD = 0.66) than viewing the video on a smartphone (M = 4.14, SD = 0.77). The elderly participants in both the smartphone group and the VR group reported a higher level of perceived telepresence (Msmartphone = 4.38, SD = 0.93; M_VR_ = 4.61, SD = 0.68) than the two groups of undergraduate students (Msmartphone = 3.90, SD = 0.48; M_VR_ = 4.37, SD = 0.63).

#### Video Preference

**FIGURE 2:**
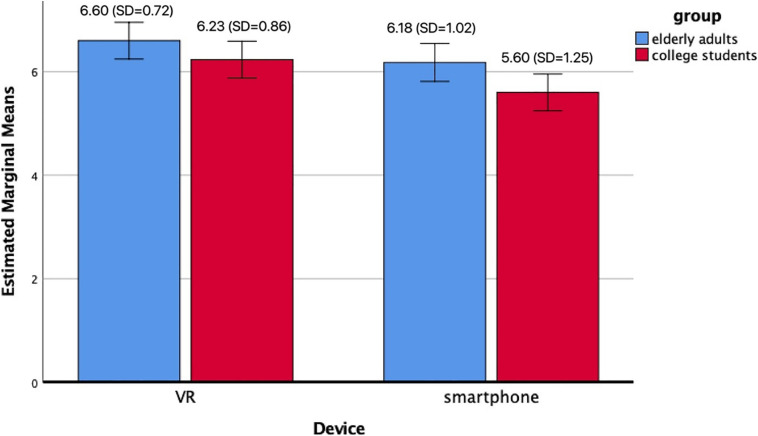
Effects of age group and device on video preference.

Then, a two-way ANOVA test was conducted to explore the difference in video preferences among the age groups and device groups. The results suggested that there were two main effects. First, the VR groups (M_VR_ = 6.42, SD = 0.809) showed more positive attitudes toward the video than the smartphone groups (Msmartphone = 5.88, SD = 1.171) regardless of age [*F*(1,114) = 6.831, *p* < 0.01)]. Second, the elderly groups (Melder = 6.4, SD = 0.897) reported more positive attitudes toward the video than the student groups (Mstudent = 5.92, SD = 1.109) regardless of the device they used (see Figure 2).

#### Viewing Experience

To investigate whether there were differences among the age groups and device groups, a two-way ANOVA was conducted. The results suggested that there was no significant difference among all groups. The participants in the different age groups showed equal preferences for the full immersive experience using a VR device and the non-immersive 360-degree video using a smartphone.

#### Mediation Effects of Age Group

**FIGURE 3:**
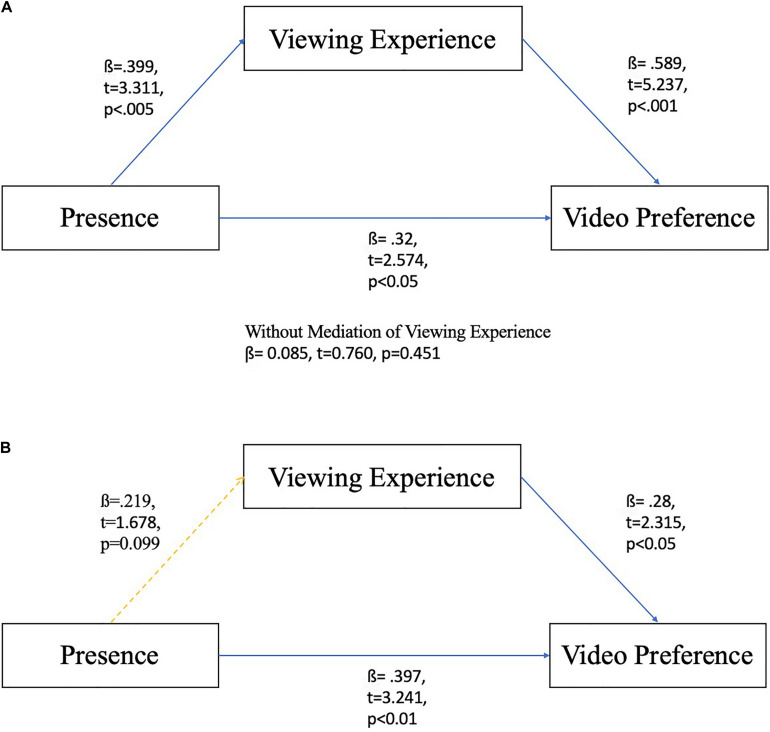
**(A)** Mediation model for young participants. **(B)** Mediation model for elderly participants.

To test the mechanism of how telepresence, video preferences and experience preferences work in different age groups, mediation models were tested. First, we tested the mediating role of watching experiences created by different devices in the relationship between perceived telepresence and preference for the video in the young student groups (see Figure 3A). Baron and Kenny’s (1986) mediation analysis was adopted to test the model. Three simple regression analyses were conducted. First, the video preference was regressed on perceived telepresence. The results showed that telepresence had a significant impact on attitudes toward the video (ß = 0.32, *t* = 2.574, *p* < 0.05); thus, the first step was supported. Then, viewing experience was regressed on telepresence. We found that telepresence had a significant positive impact on viewing experience (ß = 0.399, *t* = 3.311, *p* < 0.005). Finally, attitudes toward the video were regressed on both viewing experience and telepresence. The results showed that the impact of viewing experience on video preference was significant (ß = 0.589, *t* = 5.237, *p* < 0.001). The influence of telepresence on attitudes toward the VR video, however, was greatly reduced to a non-significant effect (ß = 0.085, *t* = 0.760, *p* = 0.451). Overall, these results demonstrated that in the college student groups, the influence of perceived telepresence on attitudes toward the video was mediated by participants’ viewing experience created by different devices.

In the elderly groups (see Figure 3B), the same procedures were used to test the same mediation model. The result of the first regression showed that the telepresence had a significant impact on attitudes toward the video (ß = 0.397, *t* = 3.241, *p* < 0.01), so step 1 was supported. For the second regression, we found that telepresence had no significant impact on viewing experience (ß = 0.219, *t* = 1.678, *p* = 0.099); therefore, step two was not supported. For the last regression, attitudes toward the video were regressed on both viewing experience (ß = 0.28, *t* = 2.315, *p* < 0.05) and telepresence (ß = 0.336, *t* = 2.77, *p* < 0.01). Both analyses suggested a significant positive impact on video preference. Because step 2 failed to be verified, the mediation effect of viewing experience between telepresence and attitude toward the video was not observed among elderly people.

In conclusion, for young students, perceived telepresence, video preferences and attitudes toward the viewing experience were all positively related. Moreover, their attitudes toward the viewing experience were affected by their perceived telepresence, which in turn influenced their attitudes toward the video. However, for the elderly participants, perceived telepresence did not necessarily lead to more positive attitudes toward the viewing experience, although telepresence and viewing experience were both positively related to video preference.

#### Purchase Intention

For the analysis related to purchasing intention toward VR devices, we only included the participants who were in VR viewing conditions. First, an independent T-test was conducted on pre-purchase intention between the elderly group and the student group. Interestingly, the results (see Table 1) demonstrated that elderly people (M = 3.63, SD = 1.088) reported a higher purchase intention toward the VR device than young students [M = 2.87, SD = 0.346, *t*(58) = 3.528, *p* < 0.05], even though only 13% of elderly people in the experiment had tried VR before. Then, another independent T-test was conducted on purchase intention after they watched the video. The result suggested an inverse trend: young people (M = 2.90, SD = 0.995) indicated higher purchase intention than elderly people [M = 2.10, SD = 0.712, *t*(58) = 3.528, *p* < 0.01] after viewing the video using the VR device.

**TABLE 1:** T-test for pre and post pruchase intention.

Variable	Group	*N*	Mean	SD	*t*	*df*	*p*
Purchase intention (pre)	Elderly adults	30	3.63	1.088	2.72	58	0.015*
	College students	30	2.87	0.346	–	–	–
Purchase intention (post)	Elderly adults	30	2.1	0.712	3.53	58	0.001***
	College students	30	2.9	0.995	–	–	–

*p < 0.05 and ***p < 0.001.

### Qualitative Results

To further understand the differences in VR experience and acceptance between the two age groups, we analyzed the in-depth interviews. Two different aspects were examined: subjective experience and VR device evaluation.

#### Subjective Experience

Regarding the subjective experiences of those who had VR viewing experiences, we found both similarities and differences between the two age groups. Generally, we extracted five themes, including (a) telepresence, (b) emotional appeal, (c) surrealness, (d) strangeness and (e) physical discomfort, from the interview materials of the elderly group. We extracted four themes from the interview materials of the student group, including (a) telepresence, (b) privacy, (c) emotional appeal, and (d) physical discomfort.

##### Telepresence

Both groups mentioned that VR gave them the sense of being there, in other words, telepresence. Meanwhile, their ways of describing the experience were quite different. The elderly group talked about their experiences based on the video content extensively and described their feelings very specifically, vividly and emotionally. The following are some quotes from the elderly participants: “At that moment, I feel like I am flying up, I am running, I am floating up. You just feel like you are there”; “My heart and my mind are totally in the video, and my attention is absolutely concentrated on it”; and “It feels like that water is pouring on you. The water is so close.” The student group was more likely to use rational and abstract words to describe the experience such as “There is a sense of reality,” “There is a sense of presence,” and “There is more sense of substitution.” The overall descriptions of telepresence by the student group seemed to be not as strong as those of the elderly group, which was consistent with what we found in the quantitative analyses that revealed that the elderly group reported a higher level of telepresence in the VR experience than the student group.

##### Emotional appeal

Most of the elderly participants reported that viewing VR videos stimulated their positive emotions. For example, they stated “I feel very pleasant after watching it”; “It feels so good to look at the kind of openness, and the kind of spectacular”; and “My ears are listening and my eyes are watching, so I forgot about the unhappiness in my heart.” There were only a few people in the student group who mentioned strong pleasure, and most students reported that during the viewing experience, their moods were quite good; however, this was not only because of the VR device but also because of the uplifting content of the video. Only two students mentioned that VR viewing greatly reduced their study-related pressure: “I felt very relaxed after I watched the VR video. I had all my confidence back at once. My emotions were totally changed.” The immersive environment created by VR technology could make people feel relaxed, forget their troubles in reality and escape for a while. Regarding this point, the elderly group and the student group had similar responses. However, a small number of elderly participants reported having a sense of “fear” and “intensity,” which rarely appeared in the student group. In general, the elderly group reported stronger emotional appeal effects than the student group for both positive and negative emotions.

##### Physical discomfort

Both the elderly group and the student group reported physical discomfort, and more people in the elderly group reported physical discomfort than in the student group. In addition to experiencing vertigo, the student group reported being uncomfortable with the shortcomings of the VR equipment and content: “The picture is not stable. Sometimes the picture will tremble. When it trembles, it makes me feel a little dizzy” and “I feel that the experience of this type of wearing is not so good; I feel the helmet always squeezing my eyes.” In addition, the elderly group reported more serious uncomfortable feelings, probably related to their health conditions. Two elderly participants dropped out of VR viewing because of vertigo: “I feel that my heart is particularly uncomfortable. The lens is pulling too fast, and the scene is rushing toward me. I feel that I can’t stand with it” and “I just think that I can’t accept it (stun), I am a little dizzy, a little uncomfortable.”

##### Privacy

Responses concerning a sense of privacy were observed only in the student group. Many student group respondents mentioned that wearing fully enclosed headgear gave them a strong sense of privacy, and they liked this exclusive personal experience; for example, students described the experience as follows: “It gives you a pure feeling of quietness and enjoyment. VR is really more shocking. You feel quiet when you are alone. It feels like this is your own time” and “When I’m in VR, I feel that I’m left alone. It’s my own world, and that feeling is really good.”

##### Surrealness and strangeness

Surrealness and strangeness were mentioned only in the elderly group. Respondents in the elderly group showed a clear sense of distinction between virtual reality and true reality in interviews. VR produced a sense of deception and unreal feelings to the elderly interviewees, and the special use of VR produced feelings of strangeness and uneasiness in some elderly interviewees. “This kind of virtual technology actually adds a barrier to emotions, which is not so intuitive and can’t be expressed intuitively”; “It’s not real, it’s fantasy.”; “I don’t think wearing goggles is human”; “VR is something that is not practical”; and “I don’t think it can be used as normal.”

#### VR Device Evaluation

In terms of device evaluation, we extracted five key themes to compare the similarities and differences regarding attitudes and opinions toward VR among the two age groups: (a) virtual telepresence, (b) technology insufficiency, (c) technology acceptance, (d) applications and (e) addiction.

##### Virtual telepresence

Most student group respondents thought that virtual telepresence could replace physical telepresence. VR technology could provide many advantages, such as saving time and money for traveling, avoiding the congestion of scenic spots, reducing the environmental pressure on scenic spots, surpassing human limitations, and recording and protecting human culture. The student responses included the following: “If I don’t have time to travel, then I can experience it with VR”; “VR could solve the problem of traveling crowds”; and “It helps to protect cultural relics, such as the exquisite and precious murals. The risk will arise, and it is difficult to protect if there are too many visiting people.” As mentioned above, the elderly group showed a clear distinction between virtual reality and true reality. Although some elderly participants also mentioned the advantages provided by VR technology, they did not think the virtual telepresence could replace the real experience.

##### Technology insufficiency

Students remarked on a series of technological insufficiencies regarding the device, such as insufficient clarity, a lack of variety in content especially made for VR devices, the unportable size of the equipment, and operational difficulty. In addition, since VR device applications are not yet widespread, some students think that it is embarrassing to use VR devices in public. Some students also mentioned some inherent flaws in VR technology, especially in terms of the interactive design. Regarding content, the excessive autonomy of VR seems to weaken the expression of the work. In addition, as a new technology, there are still some ethical issues with the use of VR and content production. For example, participants noted the following: “The device oppresses you with an oppressive feeling, which always reminds you that you are using a complicated device”; “There is a black hole at the bottom of the video, which makes you actually feel that this is not in real life”; “I feel that playing games (with VR) in public is quite awkward because you have to move around and it looks a bit funny”; “VR’s panoramic display sometimes has a negative effect on movies. Movies need to be suspended on a screen or have some lens changes, but VR can look at it from 360 degrees, which weakens the effects of expression”; and “People like me, if you don’t guide us, it is hard to mobilize our enthusiasm. I will only look in one direction. The extra 270 degrees behind me is meaningless.” Unlike the students, who had many comments on and critiques of VR, elderly participants talked more about their feelings and experiences.

##### Technology acceptance

Although many student group respondents noted the shortcomings of VR equipment, they all showed positive attitudes toward VR and believed that the deficiencies would be overcome eventually. They embraced VR technology actively and had confidence in the development and prospects of VR: “The development at the beginning may be limited to certain aspects, but I think there are a lot of things that are actively developing, and it (VR) should have a bright future”; “(Even though) I have to pay some price for VR equipment, it will make you and your descendants more proactive to embrace new technology”; and “Sometimes I doubt whether I really exist, but I am very convinced of science and technology. Even if research results are not necessarily correct, I think the new technology will bring changes to our lives, so I am willing to believe it. I think it is representative of the future.”

In contrast, many elderly group respondents thought that VR devices were too complicated to operate. The interactive function that required the body to be rotated autonomously was too complicated for some respondents. In addition, they preferred familiar media such as watching TV to maintain their emotional well-being: “Older people generally like things to be easy to operate. The procedure is complicated, and now my head is not clear. I can’t remember how to use it at all”; “I don’t have the ability to think through or manipulate it, so I still hope to have a set recommended viewing angle”; and “I can’t control it…I am afraid of operating it. I don’t usually operate (digital products).”

##### Applications

After the experiment, the elderly group respondents often voiced that they thought VR applications would be limited to entertainment and that VR devices would play content that they are used to watching (such as news shows and TV series); thus, they had no demand for other applications. These perceptions could help explain why the VR device experience was positive overall, but the elderly group did not accept VR: “The main reason is the demand; this is not needed at present”; “I am not very interested in this. We are too old, so it is useless to buy this thing”; and “I am having a satisfied life now, I don’t need this.”

Unlike the elderly group, the student participants proposed many imaginative insights into the application of VR technology. The multidomain applications mentioned included simulation driving, aerospace, virtual reality learning, business applications, and social science experiments. For example, students mentioned the following: “It can be distributed to poor areas, so that people in the mountains can see the world outside more. TV may still have some sense of distance” and “You can make a virtual space on Taobao and try on clothes like in a physical store.”

##### Addiction

Some students mentioned concerns about addiction based on their personal experience. Procrastination is a common problem among college students. Many students worried that privately owned VR equipment could reduce traveling outside and make people tend to engage in more entertainment at home: “I feel that if I really have this…with technology this advanced, I feel that I am not going to go out to play.” In the elderly group, there were such concerns as well: “Because it may make me want to continue to look at it, and then I can’t do what I want to do today. I may be addicted and like this thing too much.” Some elderly participants expressed that they were worried that their grandchildren would become addicted to VR technology.

## Discussion

VR is becoming more prevalent as a greater variety of content is available to audiences and the cost of devices is decreasing. A large amount of research has started to examine the effects of VR in different fields. However, most of these studies have only used student samples, focusing on how young people respond to this new technology and its effects on younger generations (Liu et al., 2020). It is undeniable that young people are a key audience and major users of VR, but since VR has also been widely applied in the medical health area and tourism industry in recent years (Syed-Abdul et al., 2019; Lee and Park, 2020), elderly people could become the main target. However, there are lack of studies on the experience and acceptance of VR among elderly people and how they perceive VR differently from young people. Therefore, our study provides new insights into the usage and effects of VR among the elderly.

First, our study directly compared the difference in telepresence and attitudes between young people and elderly people after having the same VR experience. The comparison results showed that elderly people reported a higher level of perceived telepresence than college students in general. This result is consistent with the qualitative data showing that the elderly people described the feeling of telepresence more frequently and used more emotional descriptions than young students in the interview. This could suggest that elderly people may be more likely to experience telepresence in VR or 360-degree videos and have more emotional responses than college students. A previous study found that the perceived level of telepresence is positively related to the attitude toward the content (Spielmann and Mantonakis, 2018). We found a consistent result that elderly people also showed more favorable attitudes toward the video than young people regardless of the device. However, we did not find any significant difference among groups regarding their attitudes toward the viewing experience. Therefore, although elderly people reported more positive attitudes toward the video than young people, this did not necessarily lead to more positive attitudes toward the viewing experience.

Second, our research adds to the body of literature on the mechanism of how telepresence, attitudes toward content and attitudes toward technology interact in different age groups. The same mediation models were tested between young students and elderly people. We found that there is a full mediation effect of viewing experience between the perceived level of telepresence and attitude toward the content in college student groups. Specifically, for young students, a higher level of telepresence can lead to a more favorable attitude toward content and in turn lead to more positive attitudes toward the content. However, this model did not work for older people. Although the level of telepresence and attitudes toward the viewing experience were both positively related to the video preference, a higher level of telepresence was not significantly related to their preferences for the viewing experience. This finding suggested that for elderly people, there should be other factors, such as perceived difficulty of using the new technology, physical discomfort and emotional responses elicited by VR, that could influence their preferences for the viewing experience in VR. For example, as shown in the qualitative analysis, some elderly people reported that they like devices that are easy to operate. VR headsets may be too complicated for elderly people for daily use. They also mentioned that they may not be able to process so much information at the same time, so some of them said they preferred only one viewpoint instead of exploring 360-degree environments by themselves. Furthermore, the feeling of “being transferred somewhere” made some elderly people feel “fearful” during watching, which could also have an impact on their attitudes toward the VR viewing experience. Physical discomfort was mentioned in both the student group and the elderly group, but dizziness was reported more frequently in the older adult group due to their health conditions. The feeling of discomfort may be one of the most important reasons why a higher level of telepresence did not lead to more positive viewing experience in the elderly group.

Finally, our study also provides some practical implications for the VR industry. Our results showed that elderly people reported a higher purchase intention toward VR devices than younger people before they watched the video; however, this trend disappeared and even was reversed after they experienced VR. Young people were more likely to want to purchase VR devices than older adults. Although elderly people reported more positive attitudes toward the video, this did not make them feel more motivated to buy the device. This finding demonstrated that elderly people tended to separate their perceptions of content and their interests in devices. As illustrated in our qualitative results, elderly people cared more about content quality, and they believed that VR devices were not necessary in their lives. The students were more likely to associate with and accept the content on the VR device.

There were several limitations to this study. First, we collected the experimental data of the elderly group in a community center instead of in a lab, which may have affected the results. Second, we only tested one video in our study and the sample size was fairly small. These problems could be addressed in future replications of our study by including more samples and more videos with different topics in the stimuli materials. Third, we did not measure the level of discomfort in the experiment as a covariate, which is an important factor related to viewing experience. Forth, our comparison of the young and elderly samples might have some confounders such as differences in income, educational background, and previous VR experience. Participants in our young group are all college students, but the elderly individuals in our study may have more diverse educational backgrounds. Additionally, our data showed that more young adults (56%) had VR related experience than elderly people (13%) in our study. Though we randomly assigned people in our study conditions, it might still affect the result of pre-purchase intention. Future studies should have more rigorous procedures for recruiting samples and include individuals’ past VR experience into consideration. Finally, gender effect was ruled out in our study design, but it can be an important factor to be explored in future studies.

## Conclusion

In the presented study, we used a mixed method approach, including both experiment and in-depth interviews, to explore the differences in experience and acceptance among the elderly and young groups. Several differences were found. First, our results showed that elderly adults reported a higher level of telepresence, more positive attitudes toward the video, and stronger emotional responses than young adults in VR viewing, but this did not necessarily lead to more positive attitudes toward the viewing experience. Second, how elderly people processed and evaluate VR content and devices were different from those of young people. Student participants’ attitudes toward the viewing experience were affected by their perceived telepresence, which in turn influenced their attitudes toward the video. However, for the elderly participants, perceived telepresence did not necessarily lead to more positive attitudes toward the viewing experience, although telepresence and viewing experience were both positively related to video preference. Third, elderly people reported a higher purchase intention toward VR devices than younger people before they watched the video and this trend was reversed after they experienced VR. Moreover, how the two age groups evaluated the viewing experience and VR device differently were discussed in the qualitative reports.

Our study directly compared the difference in telepresence and attitudes between young people and elderly people after having the same VR experience in particular. The results revealed important implications. Although elderly people reported to have higher level of telepresence and more positive attitudes toward the video than young people, this did not mean they preferred VR viewing. In fact, through in-depth interviews, elderly people reported their concerns and discomfort, and they believed VR was not necessary in their lives. It took time for the whole society, especially for the elderly, to accept a new kind of technology. However, the results and problems revealed in our study might suggest that VR is more suitable for being applied in professional areas and for purposeful use such as medical treatment and e-learning. For the elderly population, specialized VR products with easier operation and considerate content should be considered.

## Data Availability Statement

The raw data supporting the conclusions of this article will be made available by the authors, without undue reservation.

## Ethics Statement

The studies involving human participants were reviewed and approved by The Ethics Review Committee, School of Journalism and Communication, Beijing Normal University. The participants provided their written informed consent to participate in the study.

## Author Contributions

QL designed the study, wrote the draft, and contributed the most to the study. YW participated in the study design, conducted the quantitative data analysis, and reported the quantitative results. QT and ZL participated in the data collection and qualitative data analysis. All authors worked on the manuscript and critically revised it.

## Conflict of Interest

The authors declare that the research was conducted in the absence of any commercial or financial relationships that could be construed as a potential conflict of interest.
